# Hair levels of mercury, lead, and cadmium and their association with glycemic control in Egyptian children with type 1 diabetes

**DOI:** 10.1186/s12887-025-06134-1

**Published:** 2025-09-17

**Authors:** Hoda A. Atwa, Mohamed S. Hemeda, Rania M. Farh, Amany A. Khafagy, Basma M. Abdelaziz, Marwa A. Ibrahim

**Affiliations:** 1 Department of Paediatrics, Faculty of Medicine, Suez Canal University, Ismailia, Egypt; 2 Department of Forensic Medicine and Clinical Toxicology, Faculty of Medicine, Port-Said University, Port-Said, Egypt; 3 Family Medicine Department, Faculty of Medicine, Suez Canal University, Ismailia, Egypt

**Keywords:** Type 1 diabetes, Heavy metals, Lead, Mercury, Cadmium, Glycemic control, Children

## Abstract

**Background:**

Type 1 diabetes (T1D) is a chronic autoimmune condition potentially influenced by environmental factors, including exposure to heavy metals such as lead, mercury, and cadmium.

**Objective:**

This cross-sectional study (*n* = 62) was conducted between March 2020 and March 2022 to evaluate the relationship between PTEs exposure and glycemic control in Egyptian children with T1D.

**Methods:**

Hair samples were analyzed for potentially toxic elements (PTE) concentrations using ICP-MS. Clinical and environmental data, including dietary habits and parental smoking, were collected.

**Results:**

Among participants, 82.3% had elevated lead levels, and 53.2% had elevated cadmium levels. Children with poor glycemic control had significantly higher mean hair lead concentrations (39.6 mg/kg) compared to those with good control (6.3 mg/kg; *p* = 0.011; 95% CI: 25.2–40.7). A moderate positive correlation was found between lead levels and glycated hemoglobin (HbA1c) (*r* = 0.41, *p* < 0.001; 95% CI: 0.20–0.59). Mercury levels were associated with fish intake but not glycemic status. Cadmium levels were linked to parental smoking but not to HbA1c levels.

**Conclusion:**

Children with type 1 diabetes in this cohort exhibited significant levels of exposure to heavy metals, particularly lead and mercury. Elevated levels of these metals were associated with poorer glycemic control as indicated by higher HbA1c values. These findings highlight the importance of monitoring environmental exposures in pediatric diabetes management.

**Supplementary Information:**

The online version contains supplementary material available at 10.1186/s12887-025-06134-1.

## Introduction

Type 1 diabetes (T1D) is a chronic autoimmune disorder characterized by immune-mediated destruction of pancreatic beta cells, resulting in impaired insulin secretion and persistent hyperglycemia [1]. The global incidence of T1D varies widely, ranging from 1 to 40 cases per 100,000 annually. In Egypt, the estimated incidence is approximately 8 per 100,000 children under the age of 15, with rising trends in recent years [2].

While genetic predisposition is central to T1D development, environmental triggers—including infections, dietary components, and toxic exposures—may contribute to disease onset or progression. Heavy metals such as lead, mercury, and cadmium have received attention for their potential diabetogenic effects. These metals can disrupt pancreatic beta-cell function via oxidative stress, inflammation, and apoptosis. Lead exposure is known to increase reactive oxygen species and inflammatory cytokines, cadmium impairs calcium-mediated insulin secretion, and mercury interferes with immune regulation and promotes autoimmunity [3–5].

Despite increasing awareness of environmental influences on autoimmune diseases, few studies have explored the role of heavy metals in glycemic control among children with T1D, especially in low- and middle-income settings. In Egypt, fish consumption and parental smoking are two common environmental exposures linked to mercury and cadmium accumulation, respectively [6].

In environments with high levels of PTEs pollution, such as certain industrial and agricultural regions in Egypt, understanding the role of these metals in disease progression becomes particularly critical. Studies from Greater Cairo and the Nile Delta have reported elevated concentrations of lead, cadmium, and mercury in soil, vegetables, and groundwater [5, 7–9].

Several environmental factors have been implicated in influencing PTEs accumulation in children. Parental smoking, particularly paternal smoking, has been associated with elevated cadmium levels in offspring due to both direct and second-hand exposure pathways [10, 11]. Additionally, dietary habits such as frequent fish consumption have been identified as a major source of mercury exposure, especially in regions with contaminated water bodies or dietary patterns rich in seafood [12, 13]. These findings suggest that both household environmental exposure and diet may contribute significantly to children’s metal burdens, warranting further investigation within specific populations such as Egyptian children with T1D.

This study aims to evaluate the association between hair concentrations of lead, mercury, and cadmium and glycemic control in Egyptian children with T1D, to address an important research gap and inform environmental prevention strategies.

## Patients and methods

### Study design and setting

Between March 2020 to March 2022, this cross-sectional study was carried out at the Suez Canal University Hospital in Ismailia, Egypt. The Research Ethics Committee of the Faculty of Medicine, Suez Canal University (Approval No. 4106, dated 24/02/2020) granted ethical approval, and the Declaration of Helsinki Principles were also followed. All participants had their parents or legal guardians provide informed written consent before participation. Parents or legal guardians were free to withdraw from the study at any time without suffering negative consequences.

### Sample size calculation

Sample size was calculated using a power analysis based on correlation studies, aiming to detect a moderate correlation (*r* = 0.35) between PTEs levels and HbA1c with 80% power and α = 0.05. Using G*Power software, the minimum required sample was 58 participants. To account for potential dropouts or incomplete data, the final sample size was increased to 62.

### Inclusion criteria

We included children aged 2–18 years, regardless of sex, and diagnosed with T1D. We controlled glycemic levels by the specifications provided by the International Society for Pediatric and Adolescent Diabetes (ISPAD 2018) guidelines [14].

### Exclusion criteria

- Hair or scalp infections.
- Chronic illnesses other than diabetes.
- Physical disabilities.

### Data collection procedures

#### Participants underwent a detailed assessment, including

- All data were collected through structured face-to-face interviews conducted by trained pediatric residents using a standardized data collection sheet. Sociodemographic and environmental information was obtained from the child’s caregiver, while clinical details such as diabetes history and HbA1c values were verified from the participants’ medical records. Dietary history (e.g., fish consumption) and behavioral factors (e.g., pica, smoking exposure) were confirmed through repeated questioning and, when possible, cross-checked with accompanying family members. The data collection forms were reviewed for completeness and consistency by two independent investigators.
- A total of 62 children with previously diagnosed type 1 diabetes were recruited from the pediatric endocrinology outpatient clinic at Suez Canal University Hospital in Ismailia, Egypt. Participants were enrolled using a consecutive sampling technique during routine follow-up visits. Inclusion criteria included age between 6 and 16 years, diagnosis of T1D for at least one year, and no history of chronic comorbidities or acute infections. Written informed consent was obtained from the parents or legal guardians of all participants. Ethical approval for the study was obtained from the Institutional Review Board of Suez Canal University Faculty of Medicine.
- Sociodemographic data: age, gender, and socioeconomic status (assessed per El-Gilany’s socioeconomic scale) [15].
- Diabetes history: duration of diagnosis, insulin therapy, and latest HbA1c value.
- Dietary history: particularly frequency of fish consumption (e.g., canned tuna, sardines, salmon).
- Environmental exposures: including household smoking and pica behavior.
- Anthropometric data were also recorded, including height and weight, using standardized procedures.

#### Hair sample collection and analysis

Hair samples were collected from the occipital region using sterilized stainless-steel scissors. Strands were trimmed to 1.5–2 cm in length, with at least 5–10 mg of hair obtained per participant. Samples were sealed, labelled, and prepared for analysis through the following steps:

- Trimming to 0.3–0.5 cm lengths.
- Washing with ethanol (twice) and deionized water (four times).
- Oven drying at 80 °C for 10 h.
- Acid digestion using nitric, sulfuric, and perchloric acids (4:1:8 ratio).
- Quantitative transfer to a 25 ml volumetric flask for analysis.

PTEs concentrations (lead, mercury, cadmium) were measured using flame atomic absorption spectrophotometry (AAS; PerkinElmer, USA), as detailed in the laboratory quality control Sects. [16, 17].

Test results were expressed in mg/kg. Cut-off values used to define abnormal levels were:

- Mercury: >0.4 mg/kg.
- Lead: >3.0 mgsection. (((um: >0.32 mg/kg [18–20].

### Laboratory analysis and quality control

Hair samples were analyzed for lead (Pb), cadmium (Cd), and mercury (Hg) concentrations using flame atomic absorption spectrophotometry (AAS, PerkinElmer, USA). Calibration curves were constructed using standard solutions at five concentration levels for each metal (r² >0.998). The limits of detection (LOD) were 0.10 µg/g for Pb, 0.05 µg/g for Cd, and 0.08 µg/g for Hg. The accuracy and precision of the measurements were verified using certified reference materials (CRMs) (e.g., GBW07601, Human Hair Standard Reference Material, National Research Center for Certified Reference Materials, China). Intra-assay and inter-assay coefficients of variation (CV) were both below 10%. All samples were measured in duplicate, and quality control samples were run every 10 samples to monitor instrument drift.

Glycated hemoglobin (HbA1c) was measured using an automated ion-exchange high-performance liquid chromatography (HPLC) system (e.g., Bio-Rad D-10 Hemoglobin Testing System), standardized according to the National Glycohemoglobin Standardization Program (NGSP) and traceable to the International Federation of Clinical Chemistry (IFCC). Venous blood samples were collected in EDTA tubes and processed within 2 h of collection.

### Statistical analysis

The data was processed with SPSS version 22.0 (SPSS Inc., USA). Continuous variables were expressed as a means with standard deviation (SD), whereas categorical data were summarized into frequencies and percentages. The Shapiro-Wilk test was used to assess the normality of continuous variables. The independent t-test was used for comparisons involving normally distributed variables. The chi-square test was used for categorical data. For the assessment of relationships between continuous variables, Pearson’s correlation was used. Univariate regression analysis was performed to determine the predictors of glycemic control. Statistical significance was determined at a p-value less than 0.05.

Multivariable linear regression analysis was conducted to examine the independent associations between PTEs concentrations (lead, mercury, cadmium) and glycemic control as measured by HbA1c. The model adjusted for potential confounding variables including age, gender, socioeconomic class, fish consumption frequency, and parental smoking exposure. Variables were selected based on clinical relevance and significance in univariate analysis (*p* < 0.2). A p-value < 0.05 was considered statistically significant, and results were reported with 95% confidence intervals.

## Results

### Sociodemographic characteristics

The sample included 62 children with T1D aged 12.4 ± 4.25. The sample consisted of 58.1% females and 41.9% males. Middle socioeconomic status represented most participants (58.1%), while the rest (41.9%) were from low socioeconomic status. The average weight and height of the children participants were 40.73 ± 19.19 kg and 139.85 ± 20.83 cm, respectively. These baseline data provide essential context for the population under study, as well as for explaining subsequent analyses regarding environmental and clinical risk factors influencing glycemic control (Table 1).

**Table 1:** Socio-Demographic characteristics of children with T1D

Parameter	All Patients (*n* = 62)
Weight (kg)	40.73 ± 19.19
Height (cm)	139.85 ± 20.83
Gender	Male (41.9%), Female (58.1%)
Socioeconomic Class	Low (41.9%), Middle (58.1%)

Socioeconomic class was determined using a composite score based on parental education, occupation, and household income.

### Demographic characteristics by PTEs exposure

Differences in age, gender, and socioeconomic class between low and high exposure groups for mercury, lead, and cadmium are presented in Supplementary Table S1. No statistically significant differences were found in these demographic variables across exposure levels.

### Glycemic control distribution

Every study participant was categorized into two groups: with good control and fair to poor control based on their HbA1c levels. Only 25.8% (*n* = 16) of children, as stated in Table 2, managed to attain good glycemic control, whereas a considerable portion of 74.2% (*n* = 46) performed poorly, showcasing suboptimal control. The overall cohort mean HbA1c level of 7.94 ± 1.35% indicates participants are likely to be in a state of poorly controlled diabetes. This suggests more research needs to be done on the non-medical causes, which are likely affecting these children’s glycemic control’s environmental and behavioral aspects.

**Table 2:** Distribution of glycemic control among children with T1D

Glycemic Control	Frequency	Percentage
Good Control	16	25.8%
Fair to Poor Control	46	74.2%

Mean HbA1c Level: 7.94 ± 1.35%

### Environmental and behavioral risk factors

As indicated in Table S3, several possible behavioral and environmental risk factors associated with the accumulation of heavy metals were assessed. Considering mercury from fish, 66.1% of children were reported to consume fish 2–6 times a month, while 33.9% of them consumed it more than six times a month.

For the lead risk factor associated with pica behavior, the prevalence in the sample was quite high, as 79.0% of participants were reported to behave this way. Household smoking, thought to explain some of the cadmium burden, was reported for 56.5% of the population investigated. All these results point to significant exposure from numerous environmental sources of heavy metals.

### Potentially toxic elements (PTEs)Concentrations in hair samples

The data on PTEs levels in hair samples are presented in Table 3. Most of the children’s Normal Levels of Mercury Equated At 83.9% Percentage while the Average Marked At 0.099 (+/-) 0.083 mg/kg, whereas 16.1% Showed Abnormal Levels 1.04 (+/-) 1.18 mg/kg. Lead levels were especially high among those studied. A total of 82.3% of children had lead levels above the defined threshold, with a mean concentration of 40.7 ± 3.60 mg/kg, and 17.7% Had More or Less Normal Levels, 0.18 (+/-) 0.011 mg/kg. Also, Cadmium at Some Required Higher Marked 2.78 (+/-) 0.59 mg/kg, Which Was Abnormal For 53.2% of the Participants, Were Not In 46.8% Within Normal Limits 0.2 (+/-) 0.032 mg/kg. The figures illustrate the alarming prevalence of increased Pb and Cd levels in children suffering from T1D.

**Table 3:** Distribution of Mercury, Lead, and cadmium levels in hair samples of children with T1D

Element	Level	Mean ± SD	Frequency	Percentage
Mercury (mg/kg)	Normal	0.099 ± 0.083	52	83.9%
	Abnormal	1.04 ± 1.18	10	16.1%
Lead (mg/kg)	Normal	0.18 ± 0.011	11	17.7%
	Abnormal	40.7 ± 3.6	51	82.3%
Cadmium (mg/kg)	Normal	0.2 ± 0.032	29	46.8%
	Abnormal	2.78 ± 0.59	33	53.2%

### Mean HbA1c levels according to PTEs exposure categories

Table 4 presents the distribution of HbA1c levels according to PTEs exposure categories based on median splits. Children with higher lead exposure had significantly higher HbA1c levels compared to those with lower exposure (*p* = 0.003). No statistically significant differences were observed in HbA1c levels by mercury or cadmium exposure categories.

**Table 4:** Mean HbA1c levels according to PTEs exposure categories

Exposure Group	*n*	Mean HbA1c (%) ± SD	*p*-value
Lead (Pb)			
Low exposure (< median)	31	8.40 ± 1.2	
High exposure (≥ median)	31	9.22 ± 1.3	0.003
Mercury (Hg)			
Low exposure (< median)	31	8.75 ± 1.1	
High exposure (≥ median)	31	8.87 ± 1.4	0.613
Cadmium (Cd)			
Low exposure (< median)	31	8.72 ± 1.3	
High exposure (≥ median)	31	8.91 ± 1.4	0.472

Abnormal levels were defined as follows: Mercury > 1.1&nbsp;µg/g, Lead > 6&nbsp;µg/g, and Cadmium > 0.5&nbsp;µg/g.

### Correlation analysis

Pearson correlation coefficients between hair metal levels and glycemic markers (HbA1c and FBG) are presented in Supplementary Table S2. Lead showed a significant positive correlation with both HbA1c (*r* = 0.42, *p* = 0.006 after Bonferroni correction) and FBG (*r* = 0.39, *p* = 0.018), suggesting a consistent association between lead exposure and poorer glycemic control. Correlations involving mercury and cadmium were not statistically significant after correction.

### Univariate regression analysis

Univariate linear regression analysis Table S4 showed that lead levels predicted HbA1c values significantly (β = 0.427, *p* = 0.001), indicating that increased lead exposure correlates with worse glycemic control. In this model, both mercury (β = 0.206, *p* = 0.108) and cadmium (β = 0.227, *p* = 0.076) did not display statistically significant relationships with HbA1c.

### Multivariable analysis of predictors of glycemic control

To further investigate the potential influence of PTEs exposure on glycemic control, a multivariable linear regression model was developed with HbA1c as the dependent variable. Independent variables included hair levels of lead, mercury, and cadmium, as well as demographic and environmental factors: age, gender, socioeconomic class, frequency of fish consumption, and parental smoking. Table 5 presents the results of the regression model. Notably, lead concentration remained a statistically significant predictor of HbA1c levels, independent of other factors.

**Table 5:** Multivariable linear regression analysis of HbA1c levels and predictor variables among children with T1D

Predictor Variable	B Coefficient	Standard Error	95% Confidence Interval	*p*-value
Hair Led Level (mg/kg)	0.006	0.002	0.002 to 0.010	0.003
Hair Mercury Level (mg/kg)	0.210	0.180	−0.152 to 0.573	0.251
Hair Cadmium Level (mg/kg)	0.018	0.021	−0.024 to 0.060	0.390
Age (years)	−0.022	0.028	−0.078 to 0.035	0.435
Gender (Male = 1, Female = 0)	0.140	0.190	−0.240 to 0.520	0.463
Socioeconomic Class (Middle = 1)	−0.115	0.160	−0.435 to 0.205	0.473
Fish Consumption (> 6×/month = 1)	0.290	0.160	−0.030 to 0.610	0.076
Parental Smoking (Yes = 1)	0.188	0.150	−0.112 to 0.488	0.211

*Dependent variable: HbA1c (%)

*B: Unstandardized coefficient; CI: Confidence Interval.

## Discussion

The most recent study reported that children with (T1D)—especially those with poor glycemic control—demonstrated elevated levels of heavy metals such as mercury, lead, and cadmium in their hair samples. Moreover, among the three metals, only lead showed a statistically significant correlation with heightened HbA1c levels, suggesting that it may influence the T1D patients’ glycemic control.

Supporting our conclusion, a previous study conducted in Italy found no significant discrepancy regarding mercury levels between T1D patients and controls [14]. A study investigating the concentration of mercury in cord blood also did not find a statistically significant increase in the levels of T1D-affected newborns [21]. Another study suggested similar concentrations of mercury in diabetics compared to their non-diabetic counterparts [22]. On the other hand, a longitudinal investigation of toenail mercury levels in diabetic patients over 18 years found that diabetic patients had significantly higher levels than non-diabetics. This lower thyroid function study did not differentiate between T1D and T2D. A systematic review did suggest some Links between mercury exposure and diabetes, but the scope was Limited. The review focused solely on type 2 diabetes and was devoid of any evidential causation [23].

In this work, we found that elevated mercury levels were associated with higher fish consumption as well as increased HbA1c levels. These results align with a study from China where children consuming marine and freshwater fish had higher mercury concentrations [12]. Similar associations have been reported with peer studies assessing mass fish intake every week and increased mercury levels [13, 24].

In the case of lead, 82.3% of the children assessed had abnormal hair lead levels and exhibited a strong positive association with HbA1c. Moreover, lead levels were markedly higher in poorly controlled glycemic children. These outcomes are consistent with other studies, which highlighted heightened blood lead levels in diabetic adults [25]. Contrarily, some studies did not find significant differences in lead levels between T1D patients and their controls [14, 26]. Despite this, lead-induced oxidative stress is still a reasonable proposition to result in beta-cell destruction [14]. We also found that pica behaviour was strongly associated with higher lead levels, which some studies reported [27], though others did not [28]. The differing outcomes could stem from different environmental sources of lead across populations.

Cadmium levels were increased in 53.2% of participants. This was noted with other studies concerning the accumulation of cadmium in diabetics [29, 30]. In addition, a significant gender difference was noted where a greater proportion of males had abnormal cadmium levels. This could be due to greater environmental exposure in males, such as outdoor pollution and passive smoking. While it is well established that cadmium exposure impairs insulin secretion and beta-cell function of the pancreas [31], our study did not find a significant correlation between cadmium concentration and HbA1c. These outcomes are in alignment with a meta-analysis that proposed a negative or non-significant relation between diabetes and cadmium exposure in the general population [10]. On the other hand, a strong association was evident regarding abnormal cadmium levels and paternal smoking, which is consistent with previous studies [11, 32].

The discrepancies in these studies regarding cadmium can result from differences in the severity of exposure, the state of the environment, or host factors like nutritional profile or hereditary factors. Cadmium toxicity might contribute to the disruption of metabolism through other diverse means such as oxidative stress, autophagy, disrupted apoptosis pathways, and interference with crucial bio-elements [33]. As well, heavy metals like mercury with lead, and cadmium also share a common pathophysiological mechanism of oxidative stress, beta cell destructing, mediating cell destruction, which leads to the contribution and exacerbation of T1D [34].

To summarise, this research identifies a potential association between elevated lead exposure and suboptimal glycemic control in Egyptian children with T1D. While statistically significant, the cross-sectional design precludes drawing firm conclusions regarding causality.

Although not independently associated with the level of glycemic control, mercury and cadmium levels did relate to the environment through fish consumption and paternal smoking, respectively.

These findings highlight the importance of considering environmental exposures in pediatric diabetes management and suggest that efforts to monitor and mitigate lead exposure may be beneficial, pending confirmation in future longitudinal research and generating knowledge on the role of the environment in disease management. More prospective research is needed to determine the causative factors and explain the mechanisms by which heavy metals disrupt metabolic homeostasis.

### Strengths of the study

This is the first study ever conducted in the Suez Canal region that attempts to correlate the environmental exposure to heavy metals and the glycemic control in children suffering from (T1D). The research sample was robust because the authors were able to collect hair samples, which serve as a non-invasive and accurate biomarker of exposure to lead, mercury, and cadmium over a prolonged period. Furthermore, the analysts employed validated methods such as ICP-MS and implemented strict quality control measures like triplicate analysis and reference standard recovery.

Nonetheless, there are some limitations to this study worth mentioning. As a cross-sectional study, the design limits causal inference, and temporal relationships between exposure and outcome cannot be established. Additionally, the relatively small sample size, while adequate for key associations, may limit generalizability. Other important confounders, such as nutritional status or genetic predisposition, were not fully controlled.

Additional analytical work is required to establish the additional longitudinal and mechanistic frameworks needed to clarify the proposed causal pathway of how heavy metals influence T1D development and progression.

### Limitations of the study

This study has several limitations that should be acknowledged. First, its cross-sectional nature precludes establishing temporal or causal relationships between PTEs exposure and glycemic control. Second, although adjustments were made for several environmental and demographic factors, unmeasured confounders likely remain, such as detailed dietary patterns, nutritional status (e.g., iron or calcium deficiency), and genetic susceptibility to metal absorption or beta-cell damage. Third, while hair is a useful non-invasive matrix for assessing chronic metal exposure, external contamination from environmental sources or hair products, as well as individual variability in hair growth and incorporation rates, could affect measurement accuracy. Fourth, the study was conducted in a single tertiary hospital setting with a relatively small sample size, limiting the generalizability of the findings. Finally, the study did not include a healthy control group, which limits our ability to assess whether the observed levels of heavy metals are elevated compared to non-diabetic peers.

### Biological mechanisms linking heavy metals to glycemic control

The association between PTEs exposure and impaired glycemic control may be explained by several biological pathways. Lead (Pb), for instance, has been shown to induce oxidative stress, damage pancreatic β-cells, and interfere with calcium-dependent insulin secretion. Mercury (Hg) may disrupt mitochondrial function and promote pro-inflammatory cytokine production, both of which contribute to insulin resistance. Cadmium (Cd) can accumulate in the pancreas and kidney, altering glucose transporter expression and insulin receptor signalling. These mechanisms highlight how chronic exposure to toxic metals could impair glucose homeostasis, even at low concentrations. Future studies involving biomarkers of oxidative stress and inflammatory mediators could help validate these pathways in pediatric populations.

## Conclusion

This study suggests a potential association between elevated lead exposure and suboptimal glycemic control in Egyptian children with T1D. Although mercury and cadmium levels were associated with environmental factors such as fish consumption and parental smoking, respectively, no significant relationship with HbA1c was observed. These results raise the possibility that environmental exposures may influence metabolic outcomes in this population. However, given the cross-sectional design, causal interpretations cannot be made. Longitudinal studies are necessary to clarify these associations and inform appropriate public health strategies.

## Supplementary Information

Supplementary Material 1.

## Data Availability

No datasets were generated or analysed during the current study.

## Abbreviations

T1D: Type 1 Diabetes
PTEs: Potentially Toxic Elements
HbA1c: Glycated Haemoglobin
ICP-MS: Inductively Coupled Plasma Mass Spectrometry
AAS: Atomic Absorption Spectrophotometry
HPLC: High-Performance Liquid Chromatography
EDTA: Ethylenediaminetetraacetic Acid
SPSS: Statistical Package for the Social Sciences
LOD: Limit of Detection
CRMs: Certified Reference Materials
ISPAD: International Society for Pediatric and Adolescent Diabetes
