# Supplementary material for: Hair levels of mercury, lead, and cadmium and their association with glycemic control in Egyptian children with type 1 diabetes
Source: BMC Pediatr. 2025 Sep 17;25:680. doi: 10.1186/s12887-025-06134-1 (PMC12442298; doi:10.1186/s12887-025-06134-1)
Supplement: Supplementary file 1 — Supplementary Material 1. [file 12887_2025_6134_MOESM1_ESM.docx]

Supplementary Table S1. Demographic Characteristics by PTEs Exposure Levels

| Variable | Metal | Low Exposure (Mean ± SD or n%) | High Exposure (Mean ± SD or n%) | p-value |
| --- | --- | --- | --- | --- |
| Age (years) | Mercury | 10.1 ± 1.4 | 10.3 ± 1.5 | 0.641 |
| Sex (Male/Female) | Mercury | 18 / 13 | 19 / 12 | 0.712 |
| Socioeconomic Class (Low/Middle) | Mercury | 15 / 16 | 13 / 18 | 0.334 |
| Age (years) | Lead | 9.9 ± 1.3 | 10.4 ± 1.2 | 0.244 |
| Sex (Male/Female) | Lead | 16 / 15 | 15 / 16 | 0.802 |
| Socioeconomic Class (Low/Middle) | Lead | 14 / 17 | 11 / 20 | 0.109 |
| Age (years) | Cadmium | 10.2 ± 1.5 | 9.8 ± 1.4 | 0.187 |
| Sex (Male/Female) | Cadmium | 17 / 14 | 16 / 15 | 0.842 |
| Socioeconomic Class (Low/Middle) | Cadmium | 13 / 18 | 12 / 19 | 0.221 |

Supplementary Table S2. Correlation Between Hair PTEs and Glycemic Markers with 95% Confidence Intervals and Bonferroni Correction

| Variable Pair | Correlation Coefficient (r) | 95% Confidence Interval | Original p-value | Bonferroni-corrected p-value |
| --- | --- | --- | --- | --- |
| Hair Lead – HbA1c | 0.42 | 0.19 to 0.61 | 0.001 | 0.006 |
| Hair Mercury – HbA1c | 0.10 | -0.15 to 0.33 | 0.420 | 1.000 |
| Hair Cadmium – HbA1c | 0.13 | -0.11 to 0.35 | 0.284 | 1.000 |
| Hair Lead – FBG | 0.39 | 0.15 to 0.59 | 0.003 | 0.018 |
| Hair Mercury – FBG | 0.11 | -0.13 to 0.34 | 0.390 | 1.000 |
| Hair Cadmium – FBG | 0.15 | -0.09 to 0.37 | 0.215 | 1.000 |

Supplementary Table S3: Distribution of Risk Factors Related to PTEs Exposure Among Children with Type 1 Diabetes T1D

| Risk Factor | Frequency | Percentage |
| --- | --- | --- |
| Fish Consumption |  |  |
| - 2–6 times per month | 41 | 66.1% |
| - > 6 times per month | 21 | 33.9% |
| Pica (Lead Exposure) | 49 | 79.0% |
| Smoking Exposure (Cadmium) | 35 | 56.5% |

Supplementary Table S4: Univariate Linear Regression Analysis of HbA1c Levels and PTEs Concentrations in Hair Samples of Children with Type 1 Diabetes T1D

| Predictor | Unstandardized Coefficients (B) | Standard Error | Standardized Coefficients (Beta) | t-value | p-value |
| --- | --- | --- | --- | --- | --- |
| Hair  Led Level | 0.007 | 0.002 | 0.427 | 3.654 | 0.001 |
| Hair Mercury Level | 0.327 | 0.200 | 0.206 | 1.633 | 0.108 |
| Hair Cadmium Level | 0.046 | 0.026 | 0.227 | 1.807 | 0.076 |
